# An Unexpected Complication of Training: A Rare Case of Paget-Schroetter Syndrome in an Adolescent Athlete

**DOI:** 10.7759/cureus.100146

**Published:** 2025-12-26

**Authors:** Alex Abouafech, Daniel P Oar, Savannah R Chapman, Gurinder Singh, Stephen Westfall, Joe Bhagratie

**Affiliations:** 1 Orthopaedic Surgery, Lake Erie College of Osteopathic Medicine, Bradenton, USA; 2 Osteopathic Medicine, Lake Erie College of Osteopathic Medicine, Bradenton, USA; 3 Orthopaedics, Baptist Medical Center Jacksonville, Jacksonville, USA

**Keywords:** effort-induced upper extremity deep vein thrombosis, paget-schroetter syndrome, thrombo-embolic disease, venous thoracic outlet syndrome, venous thromboembolism

## Abstract

Paget-Schrotter syndrome (PSS), or exercise-induced upper-extremity deep vein thrombosis (UEDVT), is an uncommon but clinically significant phenomenon that typically affects young, otherwise healthy individuals engaged in repetitive strenuous physical activity involving the upper extremity. This case highlights a rare case of PSS in a 16-year-old female cheerleader presenting with a sudden onset of right upper-extremity swelling and cyanosis following strenuous exercise and the recent initiation of combined oral contraceptive pills. Initial evaluation with duplex ultrasonography was unremarkable, and the patient was consequently discharged with supportive care. Persistent swelling, aching, and decreased range of motion led to a repeat emergency department visit nine days later, where subsequent evaluation with duplex ultrasonography and computed tomography angiogram revealed an occlusive thrombus of the right subclavian and axillary veins. Following hospital admission with the pediatric hematology service, therapeutic anticoagulation was initiated, resulting in the gradual resolution of symptoms. This case highlights diagnostic challenges in early PSS, the interplay between strenuous exercise and estrogen-related hypercoagulability, and the importance of early detection in preventing symptom progression. By gaining a more thorough understanding of this disease process and presentation, clinicians can enhance their ability to identify and treat PSS early and effectively, thereby reducing complications and improving patient outcomes in the future.

## Introduction

Upper-extremity deep vein thrombosis (UEDVT) is a relatively rare vascular phenomenon, constituting approximately 4-10% of all deep vein thromboses [1]. Paget-Schroetter syndrome (PSS), also known as effort thrombosis, is a rare but clinically significant subtype of UEDVT with an incidence of approximately one to two cases per 100,000 people annually [2,3]. This unique diagnosis is often overlooked, resulting in a prolonged diagnostic interval and suboptimal treatment [2,3]. PSS involves thrombosis of the axillary and subclavian veins caused by mechanical compression at the thoracic outlet by structures such as the anterior and middle scalenes, first rib, and clavicle [2,3]. PSS is considered a venous variant of thoracic outlet syndrome and most often presents in young adults between ages 20 and 30, with males about twice as likely to be affected as females. This syndrome predominantly affects otherwise healthy individuals, particularly those engaging in strenuous, repetitive upper extremity activity, such as adolescent athletes [2,3]. PSS lies at the crossroads of vascular pathology and sports medicine, representing a unique disease process that warrants heightened clinical awareness and diagnostic vigilance given its potential for serious complications, including pulmonary embolism. [2,3].

In contrast to other causes of UEDVT, such as central venous catheter placement, individuals with effort-induced PSS are often symptomatic [2]. Classic symptoms of PSS in the affected extremity include significant swelling, pain, discomfort, heaviness, erythema, and dilated veins visible on inspection [4]. Occasionally, symptoms can be nonspecific, closely mimicking a simple muscle strain, delaying diagnosis and proper treatment [5]. Patients often report a specific precipitating event, often related to strenuous athletic activity involving the upper extremity, such as baseball, softball, swimming, wrestling, or gymnastics [6]. The diagnosis of PSS is first done through a thorough clinical assessment, although clinical signs of PSS have relatively low specificity [4,6]. Initial confirmatory testing is therefore completed using compression Doppler ultrasonography [7]. If diagnostic uncertainty remains, a contrast venography can also be performed; however, this diagnostic modality is more invasive and less cost-effective than standard compression ultrasound [4,6]. While magnetic resonance venography is known to have exceptional sensitivity and specificity, cost and limited availability are severely limiting factors [8].

Due to the rarity of the disease and a lack of high-quality randomized controlled trials, the optimal therapeutic approach remains up for debate [2]. Treatment is guided by patient-specific factors such as symptom duration and may include systemic fibrinolysis, local catheter-directed thrombolysis, or anticoagulation [2,4]. Thoracic outlet decompression (TOD) has become an essential component of PSS management, aimed at preventing recurrent occlusive events by eliminating the underlying anatomic obstruction [2,9,10]. TOD often involves surgical resection of the first rib, dissection of the scalene muscles, and division of the costoclavicular ligament [2,9,10].

This report highlights a rare case of PSS in a 16-year-old female athlete following strenuous physical activity. This rare case, complicated by delayed intervention, underscores the importance of early recognition and diagnostic accuracy in guiding appropriate management and improving patient outcomes. With a more comprehensive understanding of this disease process, clinicians will be better equipped to recognize and manage PSS promptly and accurately, thereby minimizing complications and optimizing patient outcomes.

## Case presentation

A 16-year-old previously healthy female presented to the emergency department with sudden-onset swelling and discoloration of the right arm that developed over several hours. She denied trauma, insect bites, fever, or systemic symptoms. The patient was a competitive cheerleader who had recently restarted combined oral contraceptive pills two weeks earlier. On examination, her right arm was diffusely swollen and mottled with cyanosis but remained warm with intact distal pulses and normal sensation. Laboratory workup revealed an elevated erythrocyte sedimentation rate (ESR), while complete blood count (CBC), comprehensive metabolic panel (CMP), and C-reactive protein (CRP) were within normal limits. Radiographs and venous ultrasound of the right arm were negative for thrombosis or fracture, and she was discharged with supportive measures and close follow-up.

Approximately three days later, the swelling persisted with aching pain rated 6/10 and intermittent purple discoloration extending to the shoulder. She was evaluated by her pediatrician and orthopedist, who suspected an inflammatory etiology and prescribed a short prednisone taper. Her oral contraceptive pills were discontinued.

**Figure 1:**
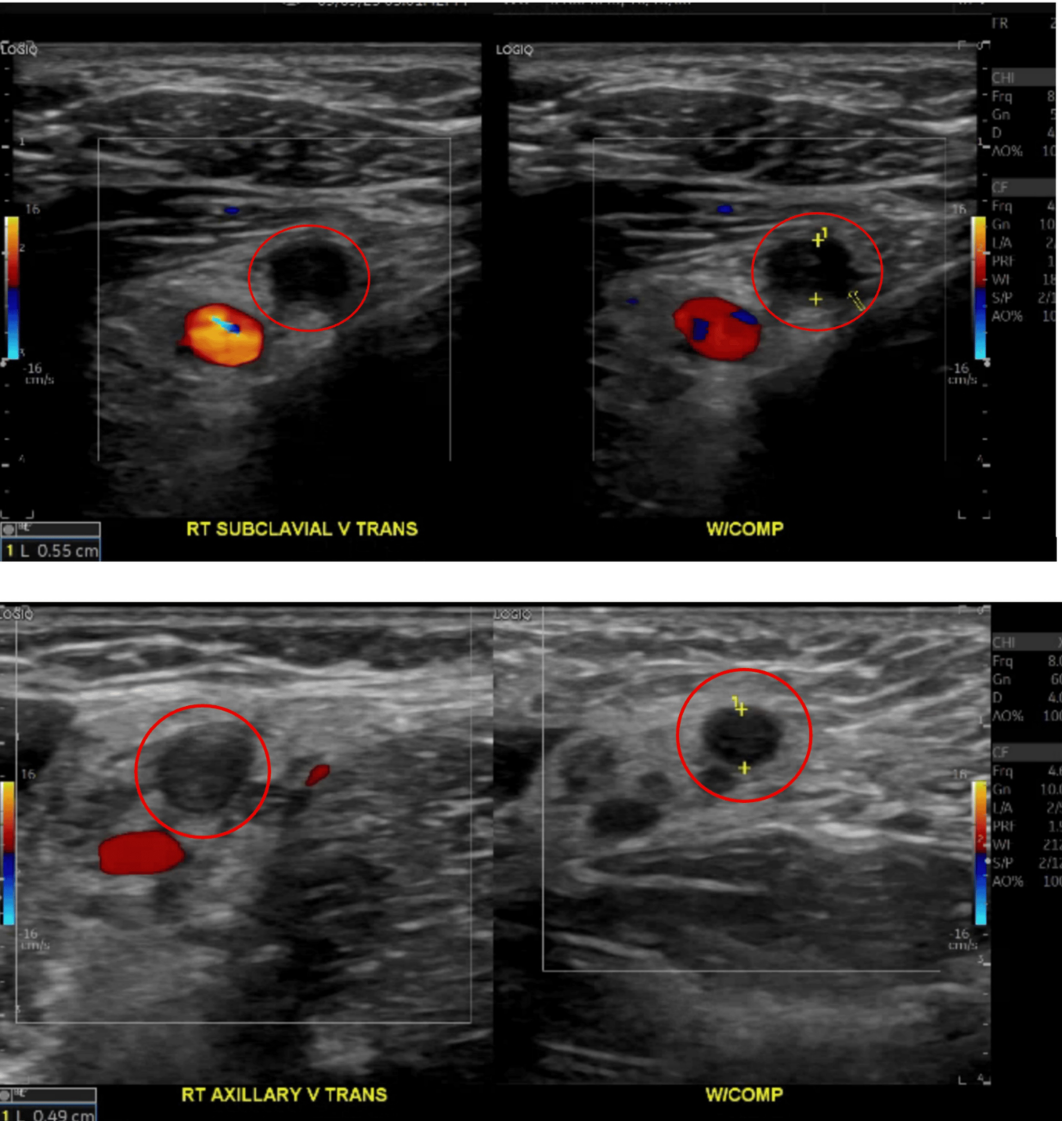
Duplex ultrasound revealing occlusive thrombosis of the right subclavian and axillary veins highlighted by the red circles with minimal compression shown on the right, signifying acute thrombosis

**Figure 2:**
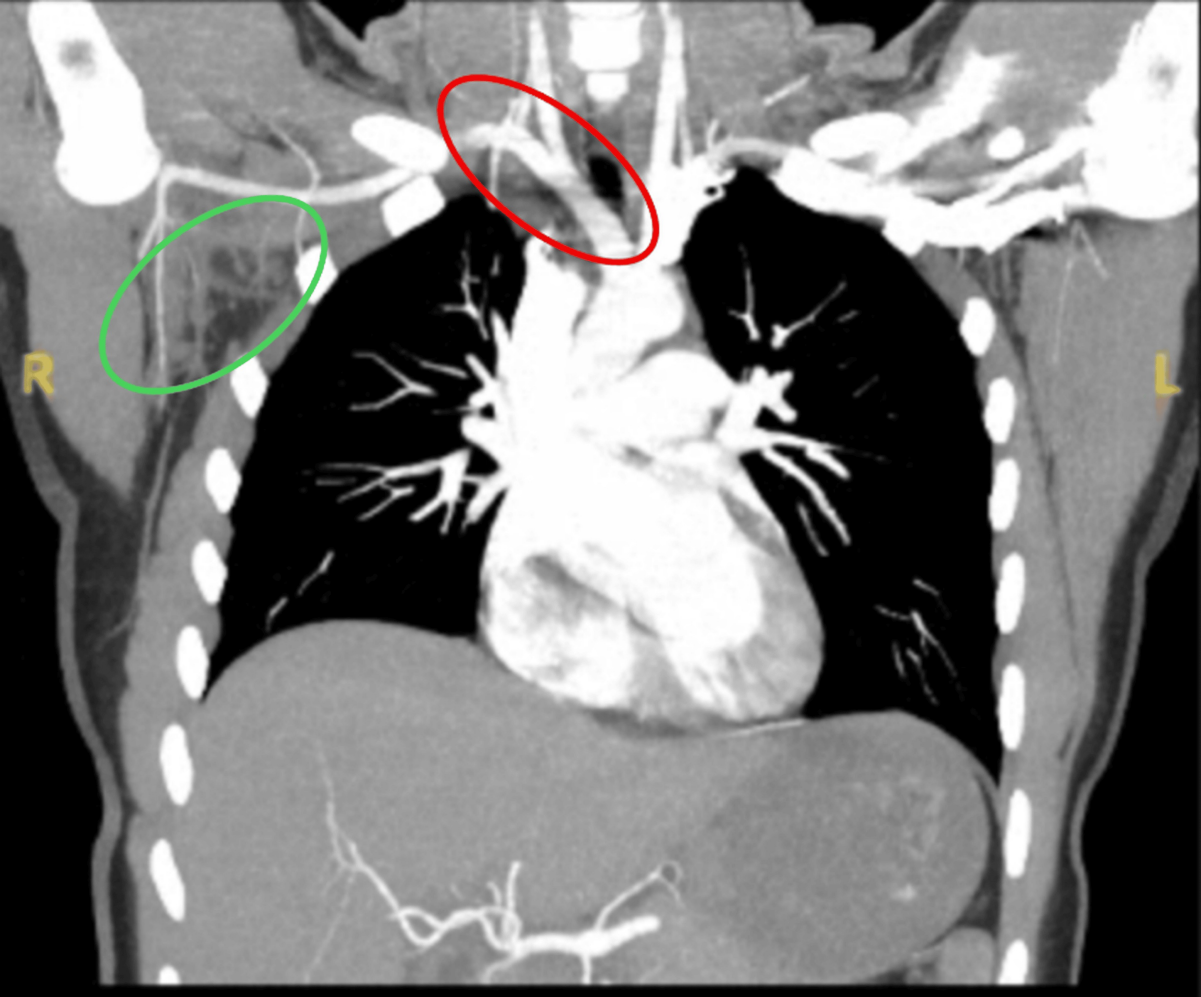
CT angiogram showing dilated, hyperdense subclavian and axillary veins indicated by the red oval with mild surrounding fat stranding indicated by the green oval, consistent with acute thrombus

One week after symptom onset, the patient returned to the emergency department with worsening swelling extending from the shoulder to the fingers, aching shoulder pain, and decreased range of motion. She continued to deny fever, chest pain, or dyspnea. Examination revealed global right-arm swelling most prominent at the shoulder, mild tenderness along the deltoid and upper arm, and reduced shoulder range of motion without erythema, warmth, or rash. Distal pulses were 2+, capillary refill was under two seconds, and sensation was intact. Laboratory studies showed an elevated ESR and D-dimer with mildly increased fibrinogen, while coagulation studies and CK were normal, as shown in Table 1. Duplex ultrasound, shown in Figure 1, revealed occlusive thrombosis of the right subclavian and axillary veins, and CT angiography of the chest, shown in Figure 2, confirmed dilated, hyperdense subclavian and axillary veins with mild surrounding fat stranding, consistent with acute thrombus. There was no pulmonary embolism, and cardiac and aortic structures were normal. The diagnosis of effort-induced subclavian-axillary vein thrombosis was made.

**Table 1:** Laboratory values upon the second emergency department visit

Parameters	Patient’s value	Reference range
ESR	58 mm/h	≤ 20 mm/h
CRP	0.7 mg/dL	≤ 1.0 mg/dL
D-dimer	776 ng/mL	< 500 ng/mL
Fibrinogen	495 mg/dL	200–400 mg/dL
Prothrombin time/international normalized ratio	11.3 s/0.97	Normal
Partial thromboplastin time	27.3 s	25–35 s
CK	42 U/L	20–200 U/L

She was transferred for inpatient pediatric hematology management. On admission, she was hemodynamically stable, with right-arm swelling and mild tenderness. She was treated with therapeutic enoxaparin injections, 60 mg every 12 hours, for several days, followed by transition to oral rivaroxaban therapy. Thrombolysis and surgical decompression were not required. She was advised to avoid nonsteroidal anti-inflammatory drugs (NSAIDs), maintain hydration, elevate the affected limb, and refrain from stunts, tumbling, or lifting, though sideline dance was permitted. She remained stable throughout hospitalization and was discharged home on oral anticoagulation with outpatient hematology follow-up arranged for one month later.

Approximately five days after discharge, she returned for a family medicine visit. She reported gradual improvement in swelling, color changes, and tingling and had completed her last enoxaparin injection before starting rivaroxaban. She noted mild fatigue, dizziness with standing, and headaches during heavy lifting, but no abnormal bleeding aside from heavier menses during hospitalization. Examination showed mild residual swelling, petechial rash near the IV site, intact perfusion, and full distal strength without clavicular or neck tenderness. She received counseling on anticoagulant precautions and nosebleed prevention. Genetic thrombophilia testing was sent for evaluation, and referrals were made to orthopedics, rheumatology, and vascular surgery to assess for possible thoracic outlet syndrome.

**Figure 3:**
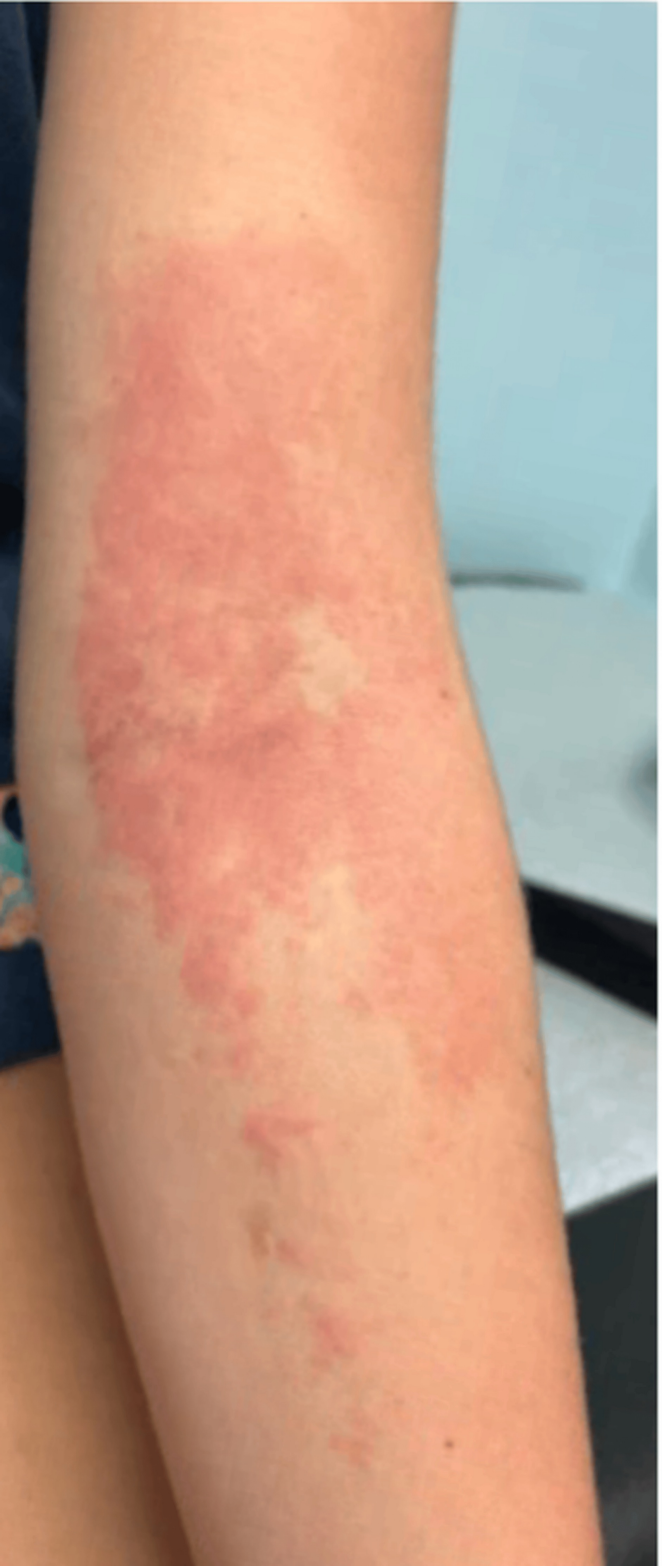
Erythematous rash on the left arm at a prior IV site

About two weeks later, during a routine follow-up, the patient developed a new erythematous rash on the left arm at a prior IV site, as shown in Figure 3. The rash began as a small patch and gradually expanded over several days, causing burning and irritation when scratched. She had intermittently used antihistamines and topical creams and reported a known latex allergy, previously triggered by medical tape. Examination revealed an erythematous rash without systemic symptoms, consistent with contact dermatitis secondary to latex or adhesive exposure. She was counseled to avoid latex products and occlusive patches, continue anticoagulation, and follow up with hematology. No recurrence of thrombosis was noted.

At a three-month follow-up, the patient reported complete resolution of swelling and pain, full recovery of shoulder mobility, and no evidence of recurrent thrombosis on duplex ultrasound. She tolerated rivaroxaban well without bleeding complications and remained off estrogen-containing contraception.

## Discussion

The case presented here highlights numerous challenges and considerations in the management of the rare PSS in an adolescent female. One of the most striking aspects of this case was the nine-day delay in both diagnosis and treatment. Although duplex Doppler ultrasound is the first-line diagnostic modality for suspected UEDVT, its sensitivity is limited, and early thrombus can evade detection on initial imaging, particularly when nonocclusive or position-dependent. Additionally, ultrasound of the upper extremity deep veins is technically challenging, especially when visualizing the subclavian vein beneath the clavicle, where acoustic shadowing can reduce diagnostic accuracy [7]. In cases such as seen here of persistent or unexplained symptoms with inconclusive imaging findings, repeat Doppler US or advanced imaging such as contrast venography or CT/MR venography should be prompted to prevent delays in diagnosis and treatment [4,6].

This case also illustrates the temporal relationship between exertion and thrombosis through Virchow’s triad of venous stasis, endothelial injury, and hypercoagulability. The patient’s repetitive overhead arm movements during cheerleading likely caused microtrauma and venous stasis, while additional mechanical compression of the subclavian vein during stunts contributed to endothelial injury [5,6]. Estrogen-induced hypercoagulability from oral contraceptive pills (OCP) use further contributed thrombotic risk [11]. The combination of mechanical stress, endothelial injury, and hematologic risk factors demonstrates how exertional activities in the setting of prothrombotic risk can precipitate thrombus formation, even in otherwise healthy, young individuals.

PSS is rare, and because of its low incidence, the optimal treatment strategy remains poorly standardized [2]. Many patients with UEDVT undergo catheter-directed thrombolysis, followed by TOD to reduce recurrence risk. Over the past two decades, this approach has become the widely accepted standard of care with multiple studies demonstrating excellent symptom resolution and reduced recurrence rates [9,10]. Current practice guidelines emphasize that anticoagulation alone is generally inadequate [9]. However, in this case, the patient achieved recovery with anticoagulation alone, initially enoxaparin, followed by rivaroxaban. This outcome supports that conservative therapy can be appropriate in carefully selected patients, particularly when diagnosis is made early, clot burden is minimal, and collateral circulation is intact, deterring more invasive interventions.

An additional element of this case involved the development of a new erythematous rash on the left arm at the site of prior IV placement during routine follow-up. The patient’s history of latex sensitivity and clinical presentation were consistent with contact dermatitis secondary to latex or adhesive exposure. Although unrelated to the thrombotic process itself, this underscores the importance of recognizing and managing complications that may occur during hospitalization or post-procedural care. If not promptly identified and treated, such issues can contribute to patient discomfort, delays in care, and unnecessary diagnostic testing. Identifying and eliminating the trigger, in this case being latex products, allowed for resolution of symptoms without interrupting anticoagulation therapy, follow-up, or continuation of care [12].

Finally, lifestyle modifications, including the avoidance of exacerbating cheerleading activities and the discontinuation of estrogen OCPs, were recommended to minimize recurrence risk [2,12]. This reflects the importance of individualized treatment planning and early intervention, particularly in healthy young individuals. The lack of adolescent-specific guidelines for PSS emphasizes the need for future research to guide diagnosis and management. By documenting our technical strategy, this report adds guidance to future clinicians facing similar case scenarios and ensures optimal patient outcomes.

## Conclusions

This case underscores the need for vigilance when evaluating adolescents with persistent unilateral arm swelling, particularly those involved in overhead athletics or recently exposed to estrogen-containing contraceptives. Because early imaging may fail to detect evolving thrombosis, clinicians should pursue repeat vascular studies when symptoms persist or progress. Timely recognition and initiation of anticoagulation are critical to minimizing the risks of embolic events and long-term post-thrombotic morbidity, often obviating the need for invasive intervention. Coordinated multidisciplinary care supports comprehensive evaluation for underlying anatomic or hypercoagulable contributors and facilitates a safe return to full activity.
